# Diffusion-weighted MRI of the spinal cord in cervical spondylotic myelopathy after instrumented fusion

**DOI:** 10.3389/fneur.2023.1172833

**Published:** 2023-05-19

**Authors:** Kevin M. Koch, Andrew S. Nencka, Andrew Klein, Marjorie Wang, Shekar Kurpad, Aditya Vedantam, Matthew Budde

**Affiliations:** ^1^Department of Radiology, Medical College of Wisconsin, Milwaukee, WI, United States; ^2^Department of Neurosurgery, Medical College of Wisconsin, Milwaukee, WI, United States

**Keywords:** cervical spondylotic myelopathy, magnetic resonance imaging, diffusion, metal artifact, spinal fusion

## Abstract

**Introduction:**

This study investigated tissue diffusion properties within the spinal cord of individuals treated for cervical spondylotic myelopathy (CSM) using post-decompression stabilization hardware. While previous research has indicated the potential of diffusion-weighted MRI (DW-MRI) markers of CSM, the metallic implants often used to stabilize the decompressed spine hamper conventional DW-MRI.

**Methods:**

Utilizing recent developments in DW-MRI metal-artifact suppression technologies, imaging data was acquired from 38 CSM study participants who had undergone instrumented fusion, as well as asymptomatic (non-instrumented) control participants. Apparent diffusion coefficients were determined in axial slice sections and split into four categories: a) instrumented levels, b) non-instrumented CSM levels, c) adjacent-segment (to instrumentation) CSM levels, and d) non-instrumented control levels. Multi-linear regression models accounting for age, sex, and body mass index were used to investigate ADC measures within each category. Furthermore, the cord diffusivity within CSM subjects was correlated with symptom scores and the duration since fusion procedures.

**Results:**

ADC measures of the spinal cord in CSM subjects were globally reduced relative to control subjects (*p* = 0.005). In addition, instrumented levels within the CSM subjects showed reduced diffusivity relative to controls (*p* = 0.003), while ADC within non-instrumented CSM levels did not statistically deviate from control levels (*p* = 0.107).

**Discussion:**

Multi-spectral DW-MRI technology can be effectively employed to evaluate cord diffusivity near fusion hardware in subjects who have undergone surgery for CSM. Leveraging this advanced technology, this study had identified significant reductions in cord diffusivity, relative to control subjects, in CSM patients treated with conventional metallic fusion instrumentation.

## 1. Introduction

Cervical spondylotic myelopathy (CSM) is the most common cause of spinal dysfunction in adults (1). This condition is the result of degeneration of the spinal cord tissue due to chronic mechanical compression of the cord. Accurate diagnosis of CSM relies on a combination of clinical symptom/function reporting and magnetic resonance imaging (MRI) (2, 3). While conventional MRI assessments can detect bulk cord pathology and cord/canal compression, it cannot provide detailed information of the microscopic or functional spinal cord. This makes CSM a challenging disease to both diagnose and prognosticate. As such, further research is necessary to develop more accurate assessment methods and to guide targeted interventions.

The dominant microscopic mechanism of cord disease in CSM is ischemia resulting from elevated levels of pressure within the cord (4). Quantitative diffusion-weighted imaging (DWI) is a an advanced MRI technique used to detect and quantify the damage cause by this ischemia, with mean diffusivity of the cervical spinal cord having been closely linked to the severity of clinical presentation and the likelihood of a positive outcome (5). It is suggested that the higher diffusivity values seen in active cases of CSM are a result of the injury-induced vasogenic edema (6). Therefore, DWI provides valuable insight into the pathology of CSM and could help in predicting the prognosis of individuals suffering from this condition (4–6).

Since compression of the spinal cord is the primary cause of CSM disease, mechanical cord decompression is the primary method of treatment. Various approaches can be used to achieve spinal cord decompression, including the insertion of metallic hardware (screws, rods, and plates) to stabilize and fuse the decompressed area of the spine. Both anterior (corpectomy or discectomy) and posterior (laminectomy) decompression procedures often involve the fusion of the levels that have been decompressed.

The frequent use of metallic stabilization hardware in CSM management can lead to artifacts in post-surgical MRI assessments, rendering them difficult to interpret (7). Conventional DW-MRI is particularly vulnerable to these artifacts due to its use of single-shot echo planar (SS-EPI) acquisition techniques (8). SS-EPI artifacts can become so severe that images become unrecognizable at instrumented levels of the cervical spine. As a result, previous DW-MRI research studies of the instrumented spinal cord have limited their diffusion measurements to areas distant from the site of instrumentation (9, 10). However, pre-clinical studies have suggested that this distant measurement approach is not as useful as directly monitoring the cord at the site of injury and instrumentation (11), motivating the need for a better approach to obtain accurate measurements.

Relative to conventional MRI technologies, Multi-Spectral Imaging (MSI) techniques offer an order-of-magnitude improvement in metal artifact reduction (12, 13). By collecting multiple spectrally-unique MRI acquisitions—known as spectral “bins”—MSI techniques reduce implant-induced artifacts and combine them to form a composite reduced-artifact image (12). To further mitigate T2*-based signal loss artifacts near metal (7, 14), MSI sequences leverage turbo/fast-spin-echo (T/FSE) pulse sequences (15). Available on several clinical vendor MRI platforms, MSI metal artifact reduction sequences offer a variety of conventional image contrast options.

The application of multispectral imaging (MSI) concepts to metal artifact-suppressed DW-MRI remains an active field of investigation. Koch et al. provided the first demonstration of MSI principles in DW-MRI (16), leveraging a fusion of 2D-MSI (17) and split-blade (18) Periodically Rotated Overlapping ParallEL Lines with Enhanced Reconstruction (PROPELLER) (19) DW techniques (20). More recently, Lee et al. (21) proposed a 3D-MSI approach by developing carefully matched MSI-specific radiofrequency pulses for diffusion sensitization. In the present report, we utilize the 2D approach of Koch et al. (16), which has recently been demonstrated in preliminary applications for spinal cord imaging (22), assessment of soft tissue pathology near total hip replacements (23), and quantitatively assessed utilizing standardized phantoms (24).

Leveraging this new technology, the present study is the first to conduct a large-cohort DW-MRI analysis of CSM patients who have undergone cord decompression and spinal fusion with metallic instrumentation. While most previous DW studies on CSM have focused on non-instrumented pre-surgical subjects, a few studies have investigated post-surgical cases (6, 25, 26). These studies, however, utilized conventional single-shot DW-MRI methods, which inhibited assessment at instrumented levels, thus excluding CSM subjects treated with commonly used metallic decompression hardware.

DW-MRI analyses of post-surgical CSM subjects treated without the use of metallic instrumentation have consistently identified substantial reductions in diffusivity associated with decompression procedures. Sato et al. (6) observed such reductions at an acute 1-week time point, but also found that these decreases in ADC persisted at a 6-month follow-up scan. Rajasekaran et al. (26) also documented pre- and post-surgical imaging exams during their study, highlighting the correlation between decreases in diffusivity and surgical outcomes. Ma et al. (25) found that mean diffusivity tended to decrease with symptom scores. Two of these studies were limited to decompression procedures that did not require metallic instrumented fusion [laminoplasty by using ceramic spacers (25) and coralline hydroxyapatite implants (25)]. The study report by Rajasekaran et al. (26) did not disclose such a limitation, but the use of single-shot echo planar techniques largely implies a lack of proximal metallic hardware.

Given the previous findings of reduced cord ADC when spinal cord decompression is performed without additional metallic stabilization hardware, the present study aims to assess if the more commonly used approaches involving such hardware will also result in a reduction of ADC relative to non-instrumented control subjects. Moreover, this study also seeks to explore whether ADC changes within the instrumented CSM cohort are affected by the instrumentation status of individual vertebral levels.

To evaluate these hypotheses, post-surgical CSM patients who underwent metallic instrumented fusion (posterior, anterior, or both) were recruited to undergo DW-MSI imaging 3–36 months after surgery. A corresponding asymptomatic non-instrumented control group was also imaged using the same protocol. ADC values of the cord were then modeled, accounting for group (control, CSM), vertebral level, instrumentation status (instrumented, non-instrumented, adjacent to instrumentation), age, body-mass index (BMI), cord area, time duration between fusion surgery and imaging exam, and the Modified Japanese Orthopedic Association Scale (mJOA) symptom score (27).

## 2. Methods

### 2.1. Study cohort

Imaging was performed on a cohort of 38 subjects with diagnosed CSM and 25 control subjects. All subjects provided written consent into a human research study protocol approved by the Institutional Review Board at the Medical College of Wisconsin.

The following inclusion criteria were utilized for recruitment of CSM subjects: (1) at least 18 years of age, (2) surgical decompression treatment of diagnosed CSM using metallic spinal fusion stabilization hardware, and (3) decompression surgery occurring between 3 and 36 months prior to the imaging session. Potential subjects with multiple separate decompression surgeries or diagnosed spinal cord conditions beyond those related to CSM were excluded. Control subjects were recruited from an adult population (greater than 18 years of old) with no known history of spinal cord injury or disease. Sex, age, body-mass-index, and mJOA checklist data elements were recorded for all subjects. In addition, the time duration between the imaging session and the subjects' decompression surgery was also recorded. Inclusion criteria for control subjects was an absence of diagnosed spinal cord injury/degeneration or history of symptoms related to the cervical spine. Control subjects were selected to eliminate statistically significant age and sex differences relative to the CSM cohort.

### 2.2. Image acquisitions

Magnetic Resonance Imaging (MRI) was performed at 3 Tesla on a 70 cm bore high-performance clinical imaging platform (GE Signa Premier, GE Healthcare, Chicago, IL, USA). A 21-channel vendor-provided head-neck-unit was utilized for signal reception. For the purposes of the present study, commercially available isotropic T1 and T2 weighted 3D-MSI (HyperMAVRIC SL, GE Healthcare, Chicago, IL, USA) (28) were utilized for morphological imaging. 3D-MSI were collected with 1.2 mm isotropic resolution, using 2 × 2 autocalibrated parallel imaging, echo times of 8/60 ms, and repetition times of 0.8/2.5 s for respective T1/T2 weighted image acquisitions.

A prototype DW-MSI sequence (16) and commercially available reduced field-of-view single-shot echo-planar DW method (FOCUS, GE Healthcare, Chicago, IL, USA) were utilized for diffusion-measurements of the spinal cord. Axially oriented slice packages of 3-5 4 mm slices (no slice gap) were collected at 3 stations throughout the cervical spinal cord, particularly focusing on levels C2-C7/T1, where the majority of instrumentation-based fused levels are found. Each slice package was oriented orthogonal to the local spinal cord axis. Both DW-MSI and DW-FOCUS images were acquired with 2.5 × 2.5 mm in plane resolution, using echo times of 56 ms and repetition times of 4 s. A single *b* = 0 image was acquired and 3 orthogonal diffusion-weighted images were acquired with a *b*-value of 600 m/*s*^2^, which was previously found to be suitable for DW imaging of the spinal cord using DW-MSI (22). In instrumented CSM subjects, only a single FOCUS-DW slice package was acquired for artifact demonstration purposes at an instrumented level.

Given the low acquisition efficiency of the DW-MSI technique and following the recommendations of Morozov et al. (29), physiological gating was not deployed in the DWI acquisitions.

### 2.3. Image processing

Post-processing of morphological and DW images was performed in Python using the open-source Spinal Cord Toolbox (SCT) (30). To begin, the 3D-MSI T1 and T2 weighted images were pre-processed using the N4 bias field algorithm (31) to remove intensity shading. Gros et al.'s (32) SCT-based deep-learning (DL) network models were then used to produce cord segmentations from the isotropic T1 and T2 weighted 3D-MSI. Subsequently, the T1 weighted image and segmentations were registered to the T2 weighted 3D-MSI. However, the presence of metal implants caused intermittent localized failures in the SCT DL-based segmentations. To resolve this issue, an algorithm was designed to repair the SCT-based segmentation estimates using both the T1 and T2 weighted segmentation estimates (summarized within Appendix 1 in Supplementary material). Finally, the cord segmentation was used for automated labeling of vertebral levels using SCT (33).

Diffusion-weighted images were analyzed using established methodology available within SCT. For each DW slice package (i.e cervical station), the T2 weighted 3D-MSI image was registered to the mean of the diffusion-weighted acquisitions using SCT's multimodal registration wrapper function (34). This transformation was then applied to the cord segmentation, and the result utilized to derive a 35 mm cropped bounding box around the cord. Motion correction was then performed on the DW image volumes using SCT's DWI motion correction (35) method. Finally, the localized cord segmentation in the DW images' coordinate space was refined by again deploying SCT's DL cord segmentation algorithm.

Apparent Diffusion Coefficients (ADC) were computed using a monoexponential model using the baseline and *b* = 600 mm/*s*^2^ diffusion-weighted images for each direction. Python pseudo-code demonstrating the cumulative post-processing steps are provided in Appendix 1 (Supplementary material).

Manual quality control of computed ADC maps and processed cord segmentations was performed by an imaging physicist on the study team. A custom-designed semi-automated interface presented each slice, as T2 weighted, DW (*b* = 0), ADC, and cord segmentation images. Options were presented to (a) exclude slice, (b) keep slice as-is, or (c) keep slice after manual re-drawing of cord segmentation. Re-drawing of segmentations was performed using the roipoly() point-wise image tracing tool in Matlab (MathWorks, Natick, MA).

Final data elements were computed after computing mean ADC measures. Mean ADC values of the cord region of interest for each slice section were stored. The cord area was also computed from the utilized segmentations, and the cord aspect ratio at each slice was computed by fitting ellipse to cord cross-sections using the methods described by Halir et al. (36). The resulting data elements for each slice were stored by subject, cohort (control or CSM), diffusion-weighted acquisition type (MSI vs. FOCUS), and vertebral level. Each vertebral level for each instrumented CSM subject was also labeled as (a) non-instrumented, (b) instrumented, or (c) adjacent segment (i.e., bordering instrumented levels).

Recent studies have demonstrated a quantitative ADC bias when comparing PROPELLER (and MSI-based PROPELLER) methods to single-shot echo planar methods (24, 37). In the present study, a cohort of control subjects was examined using matched DW-MSI and single shot EPI in order to investigate this anticipated bias in the context of the spinal cord applications. Appendix 2 (Supplementary material) reports the robust linear bias trends identified in this controlled analysis. These trends were used then to calibrate DW-MSI values against single-shot EPI, which then enables more direct comparisons with previous studies of ADC measures within CSM subjects, all of which have used single-shot echo planar methods. The details of this calibration procedure are included in Appendix 2 (Supplementary material). Notably, this DW-MSI calibration process performs a linear transform on the entire study cohort (control and CSM) and, therefore, does not affect the linear statistical analyses employed to test the study hypotheses.

### 2.4. Statistical analysis

For descriptive group comparisons between demographic categories (i.e., age, sex), Mann-Whitney U Tests were performed. Linear mixed-effects (LME) modeling approaches were then used to test and analyze the diffusivity-focused hypotheses of the study. Twelve independent models of cord ADC were computed to assess the key questions related to the study's hypotheses. These models included a combination of study data elements including age, sex, BMI, cohort (control vs. CSM), vertebral level, instrumentation of level (yes/no), adjacent segment level (yes/no), mean ADC, cord cross-sectional area, and cord aspect ratio. As there were multiple measures from axial cord sections within each subject, subject index was modeled as a random effect in all model derivations. The resulting model designs, focused questions/hypotheses of interest, source data elements, and modeled predictors are described in Appendix 3 (Supplementary material).

Models were computed in R using the lme4 library. Analysis of the computed models was performed using the mixed() function within the afex() library in R. This testing approach estimates multiple mixed models using the lme4 library and calculates χ^2^ values and hypothesis test *p*-values for all predictors. The significance level of hypothesis test results was set to *p* < 0.05.

## 3. Results

**Figure 1:**
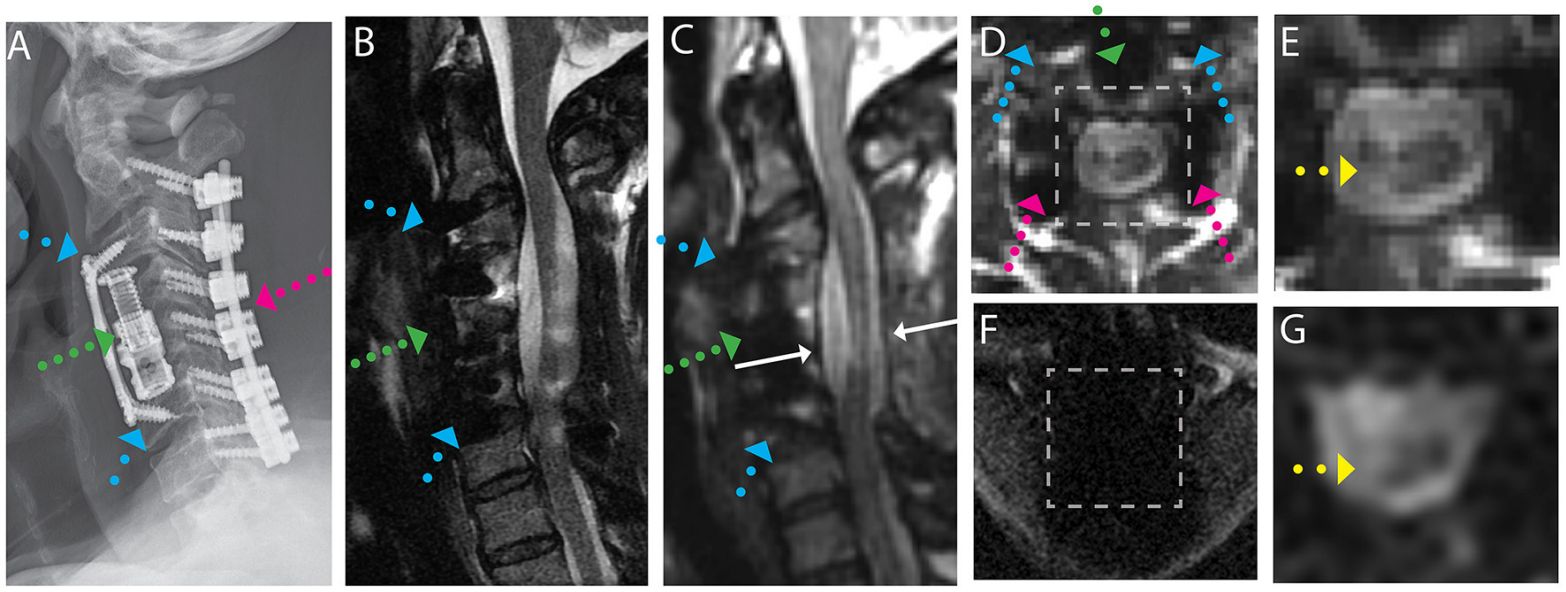
Representative images of a CSM subject with anterior and posterior fusion hardware used to treat severe cord compression with stenosis at the level of C4-C5 and congenital stenosis in the central canal. **(A)** Radiograph illustrating anterior fusion plane/screws (blue arrows), anterior interbody fusion (titanium cage, green arrow), and posterior fusion hardware (pink arrow). **(B)** Conventional MARS T2 weighted image, demonstrating substantial image distortions near the fusion hardware and throughout the cord. **(C)** Isotropic (1.2 mm) MAVRIC SL 3D-MSI T2 weighted image with minimal image artifacts. **(D)** Axial reformat of isotropic MAVRIC SL image location indicated by white arrows in **(C)**. **(E)** Zoomed MAVRIC T2w image in across box indicated in **(D)**. **(F)** Conventional single-shot *b* = 0 EPI image (FOCUS). **(G)** DW-MSI T2w (*b* = 0) within indicated box. Yellow arrows indicate region of hyperintense T2w signal within the cord.

**Figure 2:**
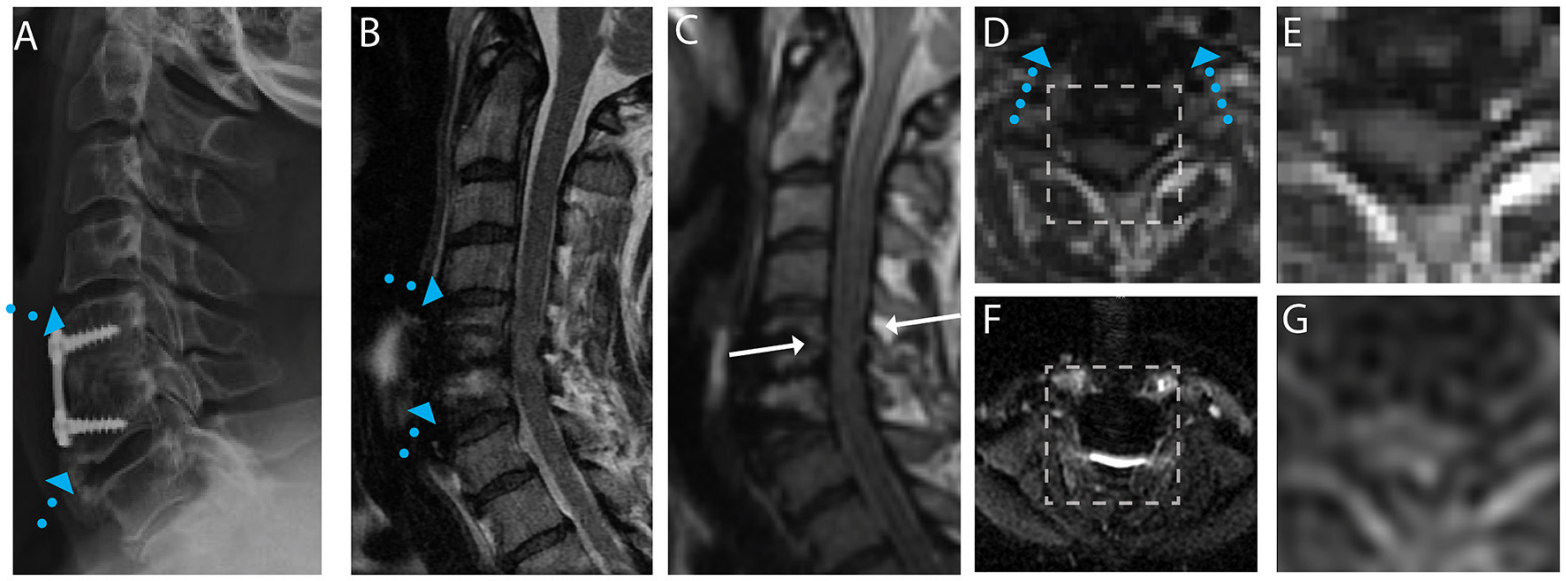
Representative images of a CSM subject with anterior fusion hardware used to treat cord compression at the level of C5-C6. **(A)** Radiograph illustrating anterior fusion plane/screws (blue arrows). **(B)** Conventional MARS T2 weighted image, demonstrating substantial image distortions near the fusion hardware. **(C)** Isotropic (1.2 mm) MAVRIC SL 3D-MSI T2 weighted image with minimal image artifacts. **(D)** Axial reformat of isotropic MAVRIC SL image location indicated by white arrows in **(C)**. **(E)** Zoomed MAVRIC T2w image in across box indicated in **(D)**. **(F)** Conventional single-shot *b* = 0 EPI image (FOCUS). **(G)** DW-MSI T2w (*b* = 0) within indicated box.

**Figure 3:**
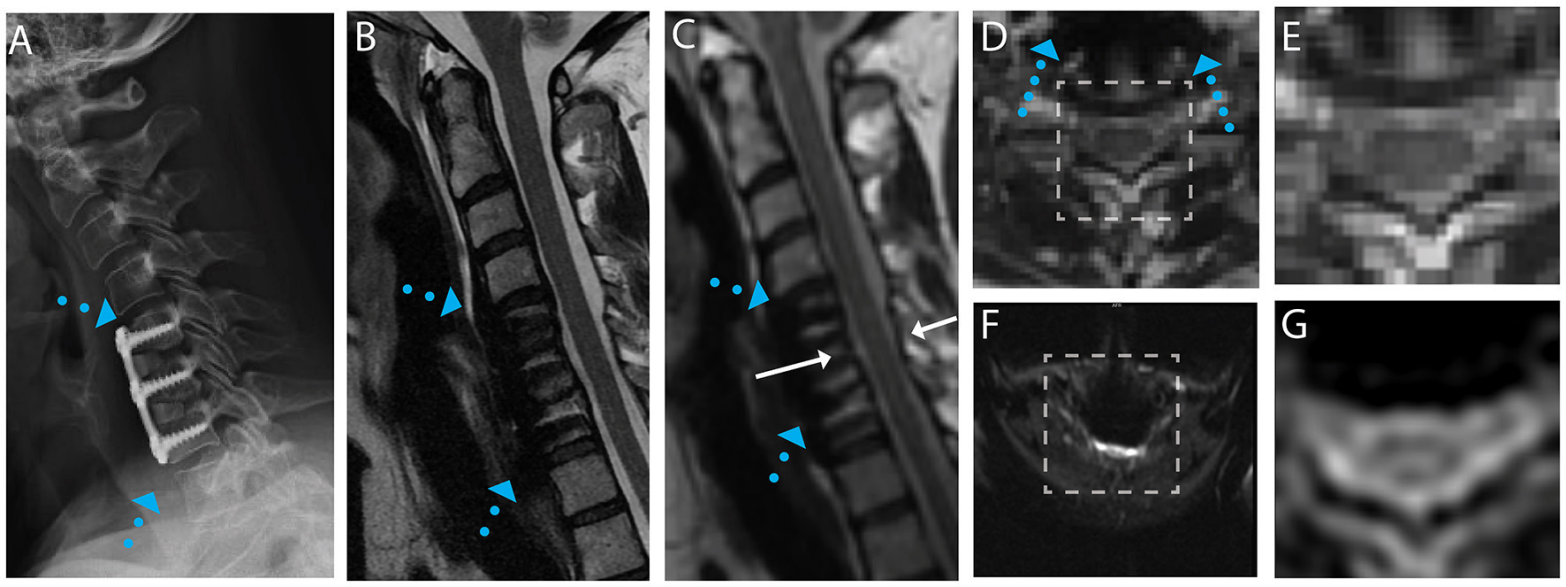
Representative images of a CSM subject with anterior fusion hardware used to treat cord compression at the level of C5-C6. **(A)** Radiograph illustrating anterior fusion plane/screws (blue arrows). **(B)** Conventional MARS T2 weighted image, demonstrating substantial image distortions near the fusion hardware. **(C)** Isotropic (1.2 mm) MAVRIC SL 3D-MSI T2 weighted image with minimal image artifacts. **(D)** Axial reformat of isotropic MAVRIC SL image location indicated by white arrows in **(C)**. **(E)** Zoomed MAVRIC T2w image in across box indicated in **(D)**. **(F)** Conventional single-shot *b* = 0 EPI image (FOCUS). **(G)** DW-MSI T2w (*b* = 0) within indicated box.

**Figure 4:**
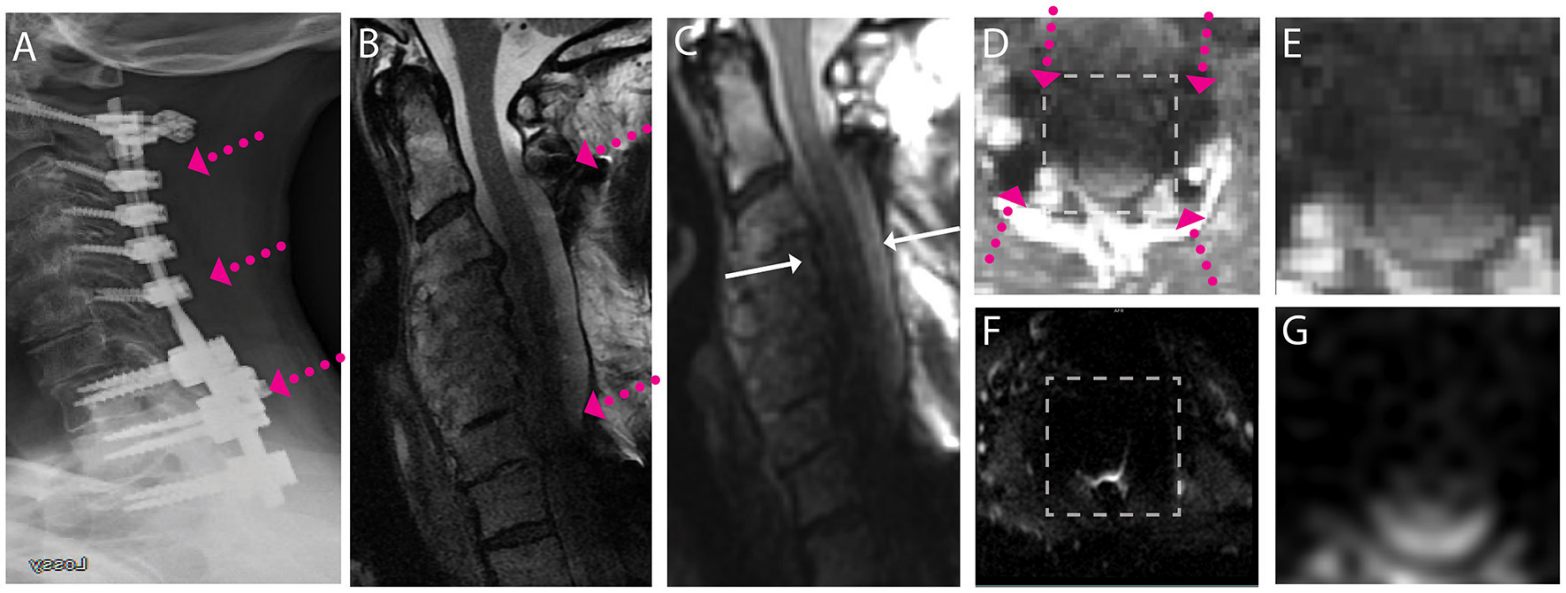
Representative images of a CSM subject with extensive posterior fusion hardware used to treat cord compression and severe stenosis across the cervical and high thoracic spine. **(A)** Radiograph illustrating anterior posterior extensive fusion plane/screws (pink arrows). **(B)** Conventional MARS T2 weighted image, demonstrating substantial image distortions across the cervical spine. **(C)** Isotropic (1.2 mm) MAVRIC SL 3D-MSI T2 weighted image with image distortions but substantial image shading due to hardware induced B_1_ field perturbations. **(D)** Axial reformat of isotropic MAVRIC SL image location indicated by white arrows in **(C)**. **(E)** Zoomed MAVRIC T2w image in across box indicated in **(D)**. **(F)** Conventional single-shot *b* = 0 EPI image (FOCUS). **(G)** DW-MSI T2w (*b* = 0) within indicated box. The DW-MSI imaging approach was unsuccessful in this scenario, due to the substantial signal degradation induced by the B_1_ shading artifact.

Exemplary sample images of instrumented fusion CSM subjects are provided in Figures 1–4. For all cases, the artifact reduction attained by the utilization of multi-spectral imaging techniques, which enables geometrically accurate morphological assessment and segmentation of the cord, is evident when comparing panels (B, conventional) and (C, 3D-MSI). The importance of multi-spectral DW methods is evidenced in panels (F, FOCUS EPI) and (G, DW-MSI), where the cord region is completely obscured by artifact in the conventional single-shot EPI (F) for all example subjects. This complete inability for FOCUS EPI to collect cord diffusion data near instrumented cord regions was observed for all study participants.

For the subject displayed within Figure 1, a local cord region of elevated T2 signal intensity (yellow arrow) is readily visible in both the axial morphological 3D-MSI (Figure 1F) and the DW-MSI (Figure 1G). The cases displayed in Figures 2, 3 provide examples of DW-MSI in the presence of anterior fusion hardware, which has less impact on cord visibility in the conventional MARS T2 image (Figures 2B, 3B), yet substantially degrades conventional FOCUS EPI DW images (Figures 2F, 3F). These cases demonstrate the ability of the DW-MSI approach to collect diffusion-weighted imaging of both the heavily compressed (Figure 2) and geometrically intact (Figure 3) cord presentations.

Finally, Figure 4 provides a scenario where even the advanced artifact mitigation of DW-MSI is unable to produce useful diffusion-weighted imaging. This subject was treated with posterior fusion hardware spanning the cervical and superior portion of the thoracic spine. The geometry and material properties of this hardware generated substantial B_1_ magnetic field perturbations, which are known to cause shading confounds, even within 3D-MSI MRI that have addressed image distortion artifacts (38). The effect of this shading is clearly evident on both the MARS (Figure 4B) and 3D-MSI T2w images (Figure 4C). The impact of this shading on DW-MSI signal integrity within the cord region is clearly evidenced in Figure 4G, where minimal cord signal is evident within the DW-MSI *b* = 0 image. This effect impacted a small number (3/38) of the study datasets and was observed only in cases with substantial superior-inferior coverage of posterior hardware. Though DW-MSI ADC maps were still capable of being collected in these cases, the number of measures that passed quality-control measures were reduced.

**Figure 5:**
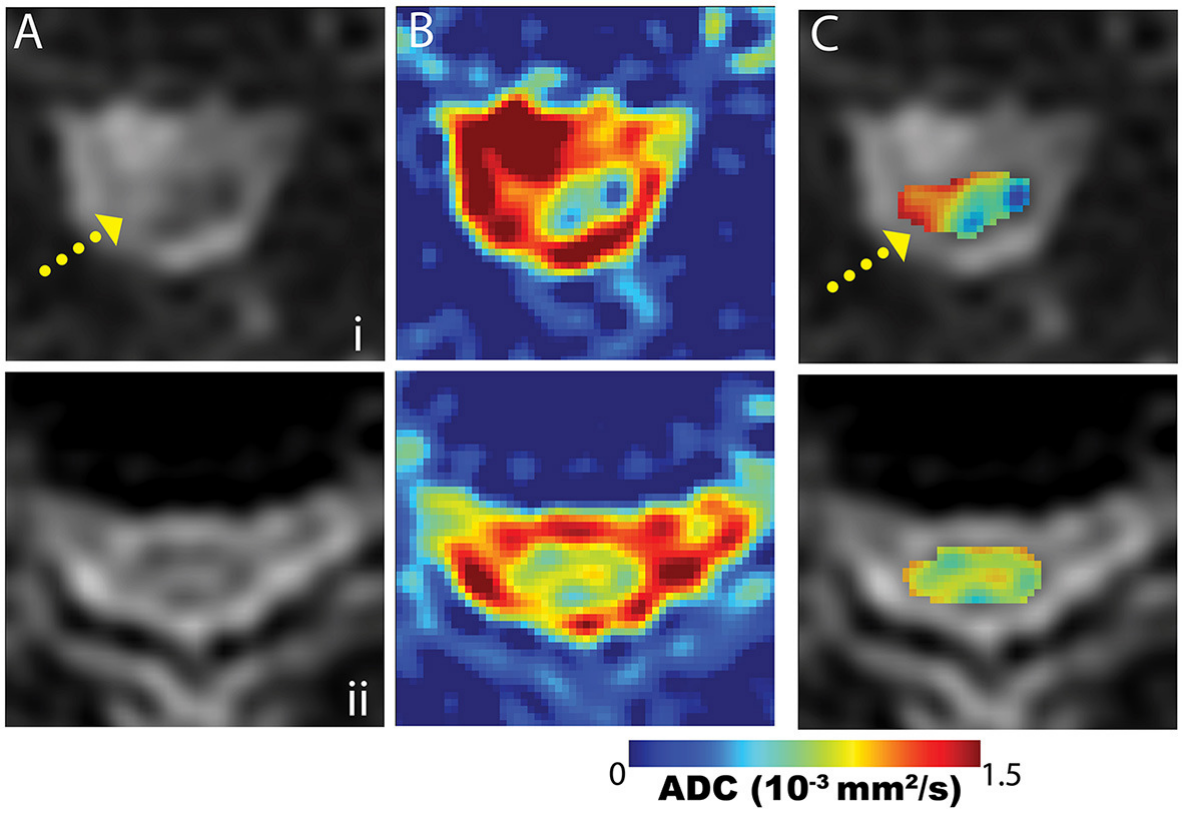
Example mean ADC maps proximal to fusion hardware from the subject cases displayed in Figure 1 (row i) and Figure 3 (row ii). Maps are presented across the spine region (column **B**) and within the cord segmentation (column **C**). The cord-segmented maps **(C)** are displayed as color maps embedded on *b* = 0 images (column **A**). The case in row (i) demonstrates an abnormal cord ADC distribution, due to the heterogeneity arising from the T2-hyperintense region of the cord (yellow arrow). Row (ii) provides a more typical cord ADC distribution when cord T2-hyperintensities are not present.

Figure 5 provides representative DW-MSI ADC maps from instrumented CSM subjects. Maps from subjects cases and axial slice sections, as illustrated in Figure 1 (row i) and Figure 3 (row ii), are provided. In these figures, column (A) displays the magnitude images with *b* = 0, column (B) features the mean ADC maps calculated across the entire spine region, and column (C) presents a fusion of the ADC map within the designated spinal cord ROI and the *b* = 0 magnitude image. Within row (i), the fused ADC map in column (C) distinctly reveals a significantly increased diffusivity in the area of hyperintense T2 weighting, suggesting the presence of spinal cord pathology. Notably, the elevated ADC region is localized, as the remainder of the cord cross-section in this slice exhibits considerably lower ADC values. Row (ii) displays a more typical ADC distribution within the spinal cord of a subject without apparent cord pathology.

**Figure 6:**
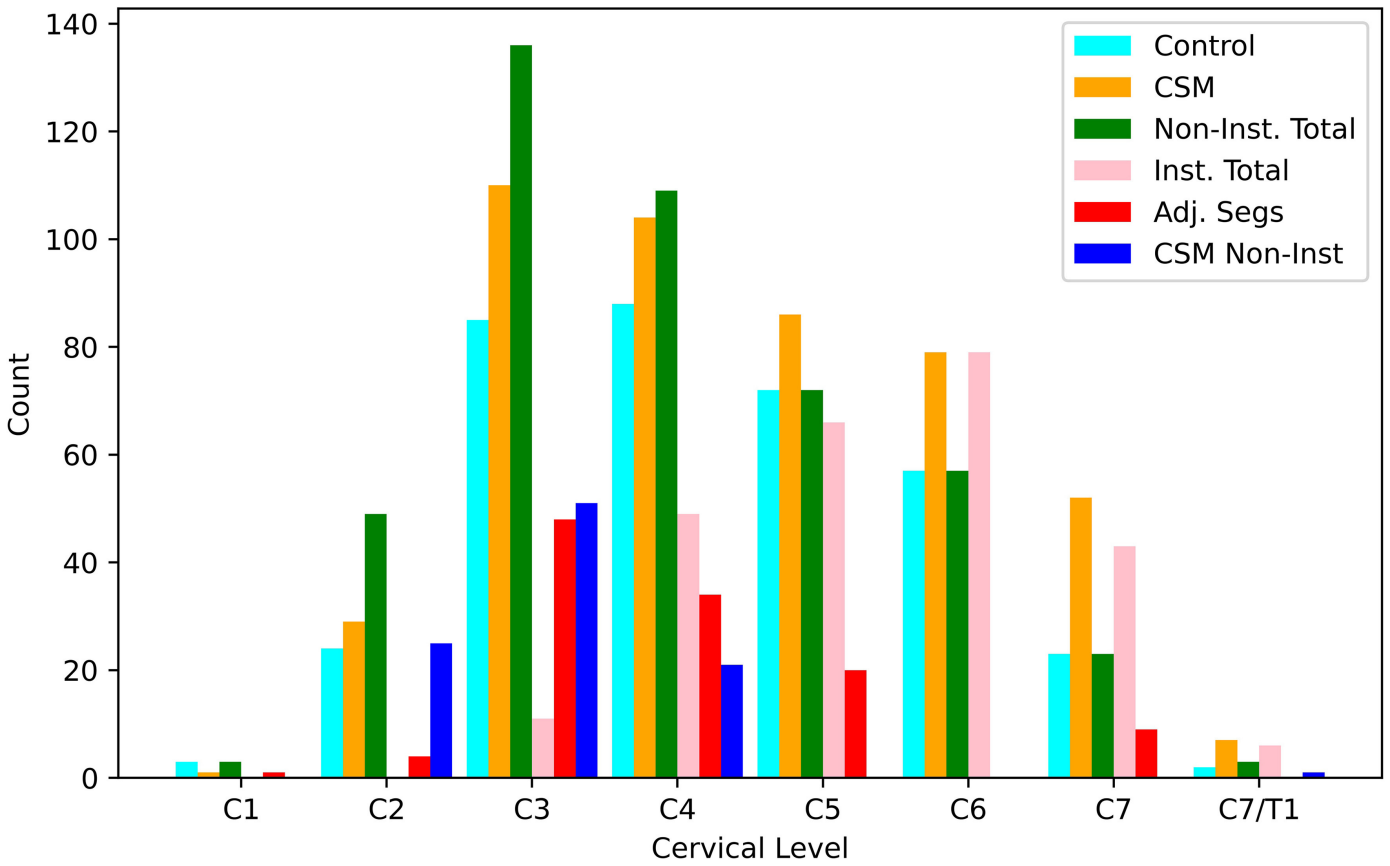
Bar-graph of transverse slice measurements of the spinal cord made at each cervical vertebral level. Counts are provided for control (*n* = 354), CSM (*n* = 468), non-instrumented levels across both cohorts (*n* = 452), instrumented levels within the CSM cohort (*n* = 254), adjacent segments within the CSM cohort (*n* = 116), and non-instrumented levels within the CSM cohort (*n* = 98).

Figure 6 illustrates the distribution of data points derived from transverse image sections of the spinal cord as a function of cervical level, cohorts (control vs. CSM), instrumented fusion status, and adjacent segment status (of each level). A total of 822 measures were collected across C1-C7/T1 in the cohorts summarized in Table 1, with the majority of the data points collected within levels C4-C7, and small distributions collected at the C1-C2 and C7/T1 levels. This sampling pattern is indicative of the predominant nature of fused levels occurring within the C4-C7 range within the study cohort. Median ADC values computed across these categories are summarized in Table 2, and reported as a function of vertebral level and analysis cohort [CSM vs. control], with the range of control and post-surgical reported mean ADC values in the calibrated DW-MSI roughly in agreement with the results reported by Sato et al. (6) and Ma et al. (25), but substantially lower than those reported by Rajasekaran et al. (26). Qualitative trends regarding ADC values as a function of instrumentation and cervical level can be observed. Raw ADC measures (prior to the DW-MSI to SS-EPI calibration procedures) are also available within Appendix 4 (Supplementary material).

**Table 1:** Cohort demographics for the study cohort of 63 subjects.

	**Male**	**Female**	**Age**	**BMI**	**mJOA**	**TPOP**
**Units**	**Count**	**Count**	**Years**	**Ratio**	**Score**	**Months**
**Desc**.			**median** ±**IQR/2**	**median** ±**IQR/2**	**median** ±**IQR/2**	**median** ±**IQR/2**
Control (*n* = 25)	12	13	55.0 ± 2.2	26.3 ± 2.7	N/A	N/A
CSM (*n* = 38)	19	19	62.0 ± 3.6	29.2 ± 1.7	15.0 ± 1.0	16.0 ± 3.4
Total	31	32	60.0 ± 2.6	28.3 ± 1.4	N/A	N/A

Continuous variables are presented as median and interquartile ranges of the distributions. TPOP, duration between fusion surgery and imaging session. None of the group demographic data elements showed statistically significant differences.

**Table 2:** ADC measures of the spinal cord, categorized by vertebral level and cohort groupings.

**Level**	**Control**	**CSM (all)**	**CSM (inst)**	**CSM (non-inst)**	**CSM (adj. seg)**	**All (non-inst)**
C1	908 ± 18 (2)	910 ± 17 (7)	907 ± 15 (6)	944 ± 0 (1)		910 ± 51 (3)
C2	946 ± 27 (3)	916 ± 0 (1)			916 ± 0 (1)	946 ± 27 (3)
C3	939 ± 6 (24)	929 ± 4 (29)		930 ± 5 (25)	924 ± 17 (4)	936 ± 4 (49)
C4	934 ± 3 (85)	926 ± 3 (110)	916 ± 9 (11)	930 ± 3 (51)	925 ± 4 (48)	934 ± 2 (136)
C5	937 ± 3 (88)	931 ± 3 (104)	931 ± 6 (49)	927 ± 6 (21)	931 ± 4 (34)	936 ± 2 (109)
C6	934 ± 3 (72)	923 ± 4 (86)	920 ± 5 (66)		927 ± 7 (20)	934 ± 3 (72)
C7	928 ± 4 (57)	916 ± 4 (79)	916 ± 4 (79)			928 ± 4 (57)
C7/T1	924 ± 6 (23)	911 ± 6 (52)	908 ± 5 (43)		933 ± 8 (9)	924 ± 6 (23)

Values are expressed in units of 10^−6^ mm^2^/s, with medians and interquartile ranges of the distributions reported. The numbers of data elements (averaged whole-cord mean ADC) within a collected slice is also reported for each result.

Table 3 provides the results of Model 1, which performed LME modeling the cord ADC against the demographic and morphological data elements available within the control cohort (sex, age, BMI, vertebral level, cord cross sectional area, and cord aspect ratio). These results showed strong inverse relationships between ADC and BMI and vertebral level. There were no significant dependencies on age, sex, cross sectional area, or aspect ratio. Given these findings, vertebral level and BMI were carried forward in all ensuing ADC models computed on the cohort data.

**Table 3:** LME results of Model 1, regressing demographic and morphological data elements against cord ADC.

**Predictor**	**β**	**χ^2^**	***P*-value**
Sex	2.88	1.14	0.286
Age	−0.06	0.17	0.676
BMI	−1.33	23.27	< 0.001
Vert. level	−2.40	43.51	< 0.001
Cross. sect. area	0.02	0.24	0.624
Cord aspect ratio	−9.04	2.08	0.149

The results of Models 2–5, which explore the effect of cohort (i.e., control vs. CSM) on cord ADC, are presented in Table 4. Following the results of Model 1, vertebral level and BMI strongly correlated with ADC. In addition, the CSM data showed a reduction in ADC relative to controls for the entire cervical cord (Model 2, *p* = 0.005) and instrumented levels (Model 3, *p* = 0.003). The non-instrumented levels (neglecting adjacent segments) did not show a statistically significant difference (Model 4, *p* = 0.107) from controls, while the adjacent segments showed a trending reduction which was slightly above the significance threshold (Model 5, *p* = 0.069).

**Table 4:** LME results of Models 2–5, investigating the impact of cohort on ADC values in different CSM level categories.

**Predictor**	**β**	**χ^2^**	***P*-value**
**Model 2: Controls vs. all CSM**			
Cohort	−6.72	7.93	0.005
BMI	−1.47	38.68	< 0.001
Vert. level	−3.62	156.83	< 0.001
**Model 3: Controls vs. inst CSM**			
Cohort	−8.50	8.49	0.004
BMI	−1.57	30.54	< 0.001
Vert. level	−3.28	80.72	< 0.001
**Model 4: Controls vs. non-inst CSM**			
Cohort	−3.35	2.60	0.107
BMI	−1.31	37.33	< 0.001
Vert. level	−2.24	47.56	< 0.001
**Model 5: Controls vs. adj. seg. CSM**			
Cohort	−4.06	3.31	0.069
BMI	−1.22	29.70	< 0.001
Vert. level	−2.06	37.47	< 0.001

ADC changes as a function of vertebral status in the CSM cohort (i.e., instrumented, non-instrumented, adjacent segment) are reported in the modeling results of Table 5. The only effect across the CSM vertebral categories was between adjacent segments and non-instrumented levels, which showed lower ADC values in adjacent segments (Model 8, Adjacent Segment Level, *p* = 0.046).

**Table 5:** LME results of Models 6–8, investigating changes in cord ADC as a function of level status (instrumented, non-instrumented, adjacent segment).

**Predictor**	**β**	**χ^2^**	***P*-value**
**Model 6: CSM non-inst. vs. inst. levels**			
Instrumented level	1.51	0.31	0.578
BMI	−1.75	17.89	< 0.001
Vert. level	−5.40	49.31	< 0.001
**Model 7: CSM inst. vs. adj seg. levels**			
Adjacent segment level	0.81	0.19	0.664
BMI	−1.76	17.66	< 0.001
Vert. level	−4.75	39.49	< 0.001
**Model 8: CSM non-inst. vs. adj. seg. levels**			
Adjacent segment level	−3.31	4.00	0.046
BMI	−1.00	9.64	0.002
Vert. level	−0.25	0.10	0.755

Finally, Table 6 provides the results of models incorporating mJOA symptom scores and post-operative duration. None of the computed models demonstrated a trend with mJOA scores. This result is unsurprising, given the lack of study participants with low mJOA scores (i.e., high degrees of symptoms). As displayed in Table 1, the CSM cohort generally demonstrated high scores with a low variance. The most revealing result in Table 6 is the connection between post-operative duration and ADC in adjacent segments (Model 12, Duration Post-Op, *p* = 0.031), which showed a reduction of ADC in adjacent segment levels as a function post-operative duration.

**Table 6:** LME results of Models 9–12, investigating changes in cord ADC as a function of mJOA score and duration post-operation in the CSM cohort.

**Predictor**	**β**	**χ^2^**	***P*-value**
**Model 9: CSM full**			
MJOA score	0.10	0.04	0.841
Duration post-op	−0.24	1.91	0.167
BMI	−1.72	20.30	< 0.001
Vert. level	−4.54	108.97	< 0.001
**Model 10: CSM inst**			
MJOA score	0.18	0.08	0.781
Duration post-op	−0.30	1.85	0.174
BMI	−2.01	16.29	< 0.001
Vert. level	−6.20	47.35	< 0.001
**Model 11: CSM non-inst**			
MJOA score	−0.08	0.02	0.880
Duration post-op	−0.25	2.67	0.102
BMI	−1.10	12.13	< 0.001
Vert. level	−1.09	2.79	0.095
**Model 12: CSM adj. seg**.			
MJOA score	−0.09	0.03	0.860
Duration post-op	−0.36	4.65	0.031
BMI	−0.94	7.74	0.005
Vert. level	1.14	1.07	0.300

## 4. Discussion

This study presents the first analysis of quantitative MRI-based diffusion measures of the spinal cord in CSM patients treated with routine metallic instrumented spinal fusion. Though previous studies have identified reductions of diffusivity in CSM patients treated with decompression surgical procedures (6, 25, 26), the use of SS-EPI DW-MRI methods has historically hindered the ability to perform measurements at levels that were decompressed using metallic instrumentation. Utilizing recently developed multi-spectral diffusion-weighted MRI techniques, the present study has revealed substantial reductions in diffusivity within the spinal cord of CSM patients treated with routine metallic fusion instrumentation.

LME regression models were utilized to probe a variety of relationships of cord diffusivity across cohorts (control and CSM) and within the CSM cohort (grouped by level instrumentation status). Due to the observed trends of cord ADC with cervical level and subject BMI, the use of multi-linear regression methods was highly warranted in this analysis. To our knowledge, this is the first spinal cord DWI study to explore BMI as a predictor of cord ADC. While other studies have identified the dependence of cord ADC with vertebral level, the strong BMI correlation observed in the present study was unexpected and warrants further investigation.

ADC measures of the spinal cord in CSM subjects were shown to be globally reduced relative to control subjects. In addition, instrumented and adjacent segment levels independently showed reduced diffusivity relative to controls. Of note, ADC within non-instrumented CSM levels did not statistically deviate from control levels. These results confirm the hypothesis that spinal fusion reduces ADC of the spinal cord, which agrees with previous findings of reduced diffusivity in decompression performed without the use of routine metallic fusion hardware (6, 25, 26).

Analysis of ADC within the CSM cohort as a function of level instrumentation status (Table 5) provided relatively little insight on changes across the instrumented cord. The only notable finding within this table was a mild decrease in diffusivity at adjacent segment levels relative to non-instrumented levels (*p* = 0.046). Though this observation is of potential interest and impact, its weak level of significance in lessens its importance in the context of other results from this study.

The correlation of adjacent segment ADC with duration post-operation is a notable finding with potential clinical impact. Adjacent segments are the greatest point of vulnerability in the fused spinal cord. The observed trend of reduced diffusivity as a function of post-operative duration indicates a potential temporal course of decompression occurring at the adjacent segments. Unfortunately, the present study design was limited to a single imaging exam, which prevented longitudinal post-surgical monitoring of diffusivity measures. Future work can leverage the results of the present analysis to perform such intervention and longitudinal monitoring studies.

Previous work by Rajasekaran et al. (26) identified a reduction on post-operative ADC in subjects that symptomatically improved after (non-instrumented) decompression procedures. As a result, a correlation between cord ADC measures and mJOA scores might have been anticipated in the present study. Though such a correlation was not found, it is important to note the limited range of mJOA scores in the recruited CSM cohort (14 ± −1), which reduced the statistical power of this sub-analysis. This limitation of the study's data complexion resulted from its generalized recruitment and analysis of post-surgical CSM subjects with metallic fusion instrumentation. The study was not scoped to look at diffusivity changes in failed surgical interventions or changes imparted by the surgical procedure itself, which would have required pre- and post-surgical imaging sessions.

Recent technical advancements have enabled metal-artifact suppressed DWI technologies (16, 21). However, these methods do not yet possess the full functionality of conventional DWI methods, introducing several additional analysis constraints. The DW-MSI technology utilized in this study (16) exhibits modest resolution and coverage capabilities, requiring multiple stations to cover the majority of the cervical spine. Moreover, DW-MSI is not yet capable of performing tensor-based acquisitions in reasonable acquisition times, and the limited resolution of the sequence prevents regional template-based measures of different tissues (i.e., gray/white matter) within the cord. Consequently, ongoing efforts are exploring technical improvements to increase slice coverage, resolution, and diffusion direction acquisitions of these sequences in order to improve the functionality of DWI in the presence of metallic hardware.

The effect of B1 shading artifacts, which is exemplified in Figure 4, is a remaining unresolved technical challenge that impacted a small number of subjects within this study (3/38). Though it impacts a small set of implant cases, it is a general remaining technical problem for MRI in the presence of metal implants that remains under active investigation.

The observed bias in DW-MSI ADC values compared to conventional EPI methods is not unique to this study. Recent investigations using standardized phantoms have identified and examined these bias trends (24, 37). Neri et al. (24) conducted comprehensive analyses and determined that this bias is dependent on the acquisition scan plane used, implying residual eddy-current effects on the DW-MSI ADC values. Due to the consistent protocol (and scan plane) applied for DW-MSI in this study, a calibration between conventional EPI and DW-MSI measures in the control cohort was achievable. The effectiveness of this approach is supported by the distinct linear calibration trend shown in Appendix 2 (Supplementary material). While this global calibration procedure does not affect the linear statistics used in the study analyses, it aligns the reported ADC values with expected levels from previous EPI-based DWI studies of CSM cohorts.

The impact of this calibration process on DW-MSI values is readily apparent. Notably, the calibrated DW-MSI values presented in Table 2 exhibit lower variance than the uncalibrated values in Appendix 4 (Supplementary material). This is due to the elimination of artificial diffusion-weighting amplification, as characterized by the slope seen in the calibration plot of Appendix 2 (Supplementary material). Although the current study was well-suited to develop and implement the presented calibration methods, the generalized utility and application of DW-MSI will necessitate further technical analyses and compensation approaches.

The value of cord DWI measurements in post-surgical CSM patients will require further analysis of the normative and pathological ADC measures across the instrumented cord. These studies should focus on measured differences pre- and post-surgery, as a function of success/failure of surgery, and longitudinally during recovery phases. Such analyses could further elucidate the importance of preliminary findings observed in the present study. In particular, the observed changes in diffusivity in adjacent relative to instrumented levels could highlight dysfunction due to longitudinal biomechanical stresses due to instrumentation.

The clinical value of DWI within post-surgical CSM patients will require further analysis of normative and pathological ADC measures across the instrumented cord. Future studies evaluating differences in DWI measurements pre- and post-surgery, as a function of the success or failure of surgery, and longitudinally during recovery phases, could provide insight into the importance of the preliminary findings observed in the current study. In particular, further examination of the observed changes in diffusivity in the adjacent levels relative to the instrumented levels could provide inform potential cord damage due to longitudinal biomechanical stresses caused by stabilization instrumentation.

In conclusion, the present study has demonstrated that DW-MRI of the spinal cord can be performed after metallic instrumented fusion management CSM. This can enable further research into the utility DW-MRI as a biomarker for CSM in both pre- and post-surgical scenarios. Observed reductions of diffusivity in the instrumented post-surgical CSM cord corroborate with previous studies of cord decompression management of CSM using no instrumentation or non-metallic instrumentation. Due to the heterogeneous conditions imparted to the post-surgical fused cord, further investigations will be required to elucidate the clinical impact and role of relative diffusivity changes in instrumented, non-instrumented, and adjacent-segment levels of the post-surgical cord.

## Author's note

Opinions, interpretations, conclusions, and recommendations are those of the authors and are not necessarily endorsed by the Department of Defense.

## Data availability statement

The raw data supporting the conclusions of this article will be made available by the authors, without undue reservation.

## Ethics statement

The studies involving human participants were reviewed and approved by Medical College of Wisconsin Institutional Review Board. The patients/participants provided their written informed consent to participate in this study.

## Author contributions

KK led the study design, analysis, and manuscript preparation. AN provided statistical assistance with study analysis and manuscript review. AK provided radiological guidance on study design and manuscript review. MW, SK, and AV provided surgical guidance on study results interpretation and manuscript review. MB provided assistance with study setup, analysis methodology, results interpretation, and manuscript review. All authors contributed to the article and approved the submitted version.
