# Supplementary material for: Diffusion-weighted MRI of the spinal cord in cervical spondylotic myelopathy after instrumented fusion
Source: Front Neurol. 2023 May 19;14:1172833. doi: 10.3389/fneur.2023.1172833 (PMC10236479; doi:10.3389/fneur.2023.1172833)
Supplement: Supplementary file 1 [file Data_Sheet_1.pdf]

Diffusion-weighted MRI of the spinal cord in cervical spondylotic myelopathy after instrumented fusion: Supplemental Material

Appendix 1: Spinal-Cord Toolbox Post-Processing Psuedo-Code [python]

MORPHOLOGICAL PROCESSING

Cord Segmentation

```
sct_deepseg_sc -i T2.nii -c T2
sct_deepseg_sc -i T1.nii -c T1
```

T1/T2 Registration/Alighment

```
sct_register_multimodal -i T1.nii -d T2.nii -identity 1 -x nn
sct_apply_transfo -i T1_seg.nii -d T2.nii -w (above) -o T1seg2T2.nii
sct_apply_transfo -i T1.nii -d T2.nii -w (above) -o T12T2.nii
```

Vertebral Leveling

```
sct_label_vertebrae -i T2.nii -s T2_seg.nii -c T2
```

CORD SEGMENTATION REFINEMENT

Begin by taking union of T1 and T2 deep-learning segmentation masks
Compute connected components for each axial slice through cord for T2 based segmentation

```
ccFilter = sitk.ConnectedComponentImageFilter()
```

Build edges in each axial slice of connected component mask

```
sitk.CannyEdgeDetection()
```

Compute a bounding box to extract combined T1 and T2 segmentation union edge

```
lsif = sitk.LabelShapeStatisticsImageFilter()
boundingBox = lsif.GetBoundingBox(1)
```

Perform segmented radial basis function fitting on bounded union segmentation
Use radial basis function library (rbf) to fit surface of T1 and T2 segmentation boundary union.

```
npSmooth = 5
[x,y,z] = Rbf(points,npSmooth)
```

Perform independent fits within each of 4 axial sectioned regions of the cord
Do this within 2 interleaves across cervical cord and take union of interleaves to get final segmentation

DIFFUSION PROCESSING

Compute mean DWI image

```
sct_maths -i DWI.nii -mean t -o dmn.nii
```

Register T2 and T2-based segmentation to DWI

```
sct_register_multimodal -i T2.nii -d dmn.nii -identity 1 -x nn
sct_apply_transfo -i T2_seg.nii  -d dmn.nii -w (above) -o segReg.nii
```

Create local crop mask around cord and crop DWI image

```
sct_create_mask -i dmn.nii -p centerline,segReg.nii -size 35mm
sct_crop_image -i DWI.nii -m mask_dmn.nii -o dmri_crop.nii
sct_crop_image -i T2_reg.nii -m mask_dmn.nii -o T2_crop.nii
```

Run motion correction on DWI series

```
sct_dmri_moco -i dmri_crop.nii -bvec
```

Re-compute cord segmentation on cropped DWI mean image

```
sct_deepseg_sc -i dmri_crop_moco_b0_mean.nii -c T2
```

Appendix 2: DW-MSI Calibration Against Single-Shot EPI DWI

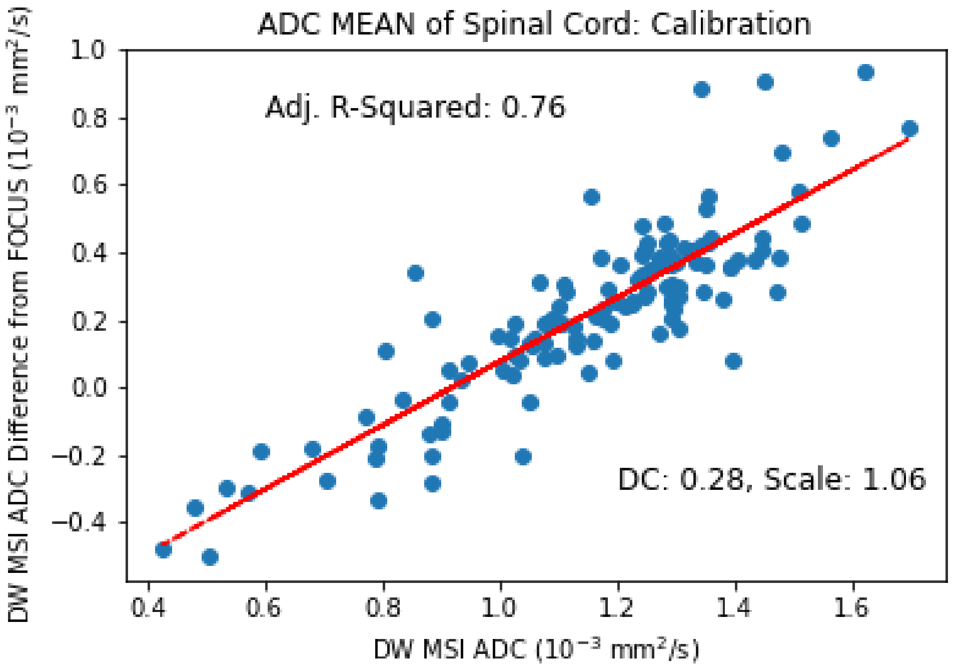

Figure 1: Fig. A2.1: Plot of DW-MSI error relative to Single-Shot EPI DWI (FOCUS) for mean ADC metric, along with computed regression trend and resulting calibration coefficients. Note the bias (offset) and slope (diffusion scaling) that is revealed from the calibration.

Calibration of DW-MSI was performed by computing the linear trend between the difference of paired EPI and DW-MSI ADC values (ADC_{EPI}, ADC_{MSI}) in the control cohort:

$$ADC_{MSI} - ADC_{EPI} = \beta ADC_{MSI} + \alpha.$$

The calibrated DW-MSI values, ADC_{MSI}^{cal} are then computed using these coefficients, according to:

$$ADC_{MSI}^{Cal} = ADC_{MSI}(1 - \beta) - \alpha.$$

Appendix 3: Descriptions of computed LME Models

1. General: Which demographic, clinical, and basic morphological data elements impact cord diffusion?
   - Source data: All controls
   - Predictors: All control data elements
2. Cohort Differences: Is the whole cord ADC different between control and instrumented CSM cohorts ?
   - Source data: All study subjects
   - Predictors: All elements with statistically significant results in Model 1, along with the cohort label for each measurement.
3. Cohort Differences: Is ADC different within instrumented CSM levels compared to controls?
   - Source data: Instrumented CSM levels and control subjects
   - Predictors: All elements with statistically significant results in Model 1, along with the cohort label for each measurement.
4. Cohort Differences: Is ADC different within non-instrumented (non-adjacent segment) levels in CSM levels compared to controls?
   - Source data: Non-instrumented (non-adjacent-segment) CSM levels and control subjects
   - Predictors: All elements with statistically significant results in Model 1, along with the cohort label for each measurement.
5. Cohort Differences Is ADC different within adjacent segment levels in CSM levels compared to controls?
   - Source data: Adjacent segment CSM levels and control subjects
   - Predictors: All elements with statistically significant results in Model 1, along with the cohort label for each measurement.
6. Intra-CSM Categories: Is ADC different within instrumented vs non-instrumented (not including adjacent segment) levels of CSM subjects?
   - Source data: Instrumented and non-instrumented (non-adjacent segment) levels in CSM
   - Predictors: All elements with statistically significant results in Model 1, along with the instrumented level indicator for each measurement.
7. Intra-CSM Categories: Is ADC different within instrumented vs adjacent segment levels of CSM subjects?
   - Source data: Instrumented and adjacent segment levels in CSM
   - Predictors: All elements with statistically significant results in Model 1, along with the instrumented level indicator for each measurement.
8. Intra-CSM Categories: Is ADC different within non-instrumented (non-adjacent) vs adjacent segment levels of CSM subjects?
   - Source data: All non-instrumented CSM levels
   - Predictors: All elements with statistically significant results in Model 1, along with the adjacent segment level indicator for each measurement.

9. CSM Symptom Correlations: Does whole-cord ADC correlate with mJOA scores or duration since the fusion procedure?
    - Source data: CSM cohort
    - Predictors: All elements with statistically significant results in Model 1, mJOA score, duration since fusion procedure.
10. CSM Symptom Correlations: Does instrumented level ADC correlate with mJOA scores or duration since the fusion procedure?
    - Source data: Instrumented levels of CSM cohort
    - Predictors: All elements with statistically significant results in Model 1, mJOA score, duration since fusion procedure.
11. CSM Symptom Correlations: Does non-instrumented (non-adjacent) level ADC correlate with mJOA scores or duration since the fusion procedure?
    - Source data: Non-instrumented (non-adjacent segment) levels of CSM cohort
    - Predictors: All elements with statistically significant results in Model 1, mJOA score, duration since fusion procedure.
12. CSM Symptom Correlations: Does adjacent segment level ADC correlate with mJOA scores or duration since the fusion procedure?
    - Source data: Adjacent segment levels of CSM cohort
    - Predictors: All elements with statistically significant results in Model 1, mJOA score, duration since fusion procedure.

Appendix 4: Raw (uncalibrated) DW-MSI ADC Values

	Control	CSM (All)	CSM (Inst)	CSM (Non-Inst)	CSM (Adj. Seg)	All (Non-Inst)
C1	733 ± 340 (2)	757 ± 308 (7)	706 ± 268 (6)	1382 ± 0 (1)		760 ± 933 (3)
C2	1420 ± 488 (3)	879 ± 0 (1)			879 ± 0 (1)	1420 ± 488 (3)
C3	1290 ± 110 (24)	1117 ± 81 (29)		1123 ± 85 (25)	1023 ± 319 (4)	1237 ± 68 (49)
C4	1211 ± 50 (85)	1054 ± 48 (110)	871 ± 166 (11)	1126 ± 62 (51)	1038 ± 81 (48)	1201 ± 39 (136)
C5	1256 ± 49 (88)	1154 ± 61 (104)	1155 ± 114 (49)	1073 ± 116 (21)	1152 ± 68 (34)	1250 ± 45 (109)
C6	1199 ± 60 (72)	997 ± 75 (86)	941 ± 90 (66)		1086 ± 129 (20)	1199 ± 60 (72)
C7	1100 ± 69 (57)	876 ± 81 (79)	876 ± 81 (79)			1100 ± 69 (57)
C7/T1	1026 ± 117 (23)	789 ± 103 (52)	721 ± 100 (43)		1185 ± 145 (9)	1026 ± 117 (23)

ADC measures of the spinal cord using uncalibrated DW-MSI, categorized by vertebral level and cohort groupings. Values are expressed in units of 10^{-6} mm^2/s, with medians and interquartile ranges of the distributions reported. The numbers of data elements (averaged whole-cord mean ADC) within a collected slice is also reported for each result.
